# Supplementary material for: Dietary Lactoferrin Supplementation Improves Growth Performance and Intestinal Health of Juvenile Orange-Spotted Groupers (Epinephelus coioides)
Source: Metabolites. 2022 Sep 28;12(10):915. doi: 10.3390/metabo12100915 (PMC9607261; doi:10.3390/metabo12100915)
Supplement: Supplementary file 1 [file metabolites-12-00915-s001.zip › Supplementary material.pdf]

Supplementary material

Table S1: MetaStat analysis of the abundance of DI bacterial phyla and genera (× 10^{-4}) of juvenile orange-spotted grouper in a 56-d feeding period.

	Diets				
	FM	SBM60	LF2	LF6	LF10
Phylum					
Firmicutes	3757.68±418.75	4505.65±781.74	2259.32±1420.22	4043.05±775.32	4424.59±491.46
Proteobacteria	2738.07±766.75	2313.86±920.10	5638.20±2435.90	2781.97±1410.64	2373.36±532.88
Bacteroidetes	2775.85±530.90	2493.30±505.40	1603.40±1069.54	2893.40±664.29	2901.57±83.84
Fusobacteria	34.49±17.62	487.82±352.13	356.26±345.18	43.62±42.49	35.04±9.47
Spirochaetae	456.43±438.31	18.82±3.93	2.75±1.84	79.96±53.54	44.72±11.97
Genus					
Photobacterium	1896.05±632.95	1117.64±704.11	3525.27±2715.36	1758.45±1508.44	1159.47±435.72
Selenomonas_1	1372.16±64.07	1304.08±301.40	532.34±320.71	1175.27±293.29	1478.02±272.20
Prevotella_1	831.10±152.96	778.75±207.82	511.73±305.10	879.39±303.93	920.75±171.80
Vibrio	68.29±66.84	371.93±369.97	1669.90±1644.07	76.05±60.73	171.74±109.18
Rikenellaceae_RC9_gut_group	324.46±79.78	380.86±114.12	287.02±210.38	667.19±220.78	459.59±111.90
Curvibacter	296.70±59.07	279.94±142.20	70.35±23.07	324.18±133.27	396.59±125.58
Christensenellaceae_R-7_group	311.61±26.64	242.71±78.45	57.84±15.02	288.05±54.04	244.70±42.70
Veillonellaceae_UCG-001	157.80±6.14	242.36±74.74	105.18±49.46	273.48±66.19	330.02±94.82
Anaerovibrio	151.00±40.18	220.31±12.55	139.94±83.43	199.57±21.26	340.19±48.37
Succiniclasticum	159.93±34.58	211.24±67.49	145.78±99.86	203.41±75.71	237.14±60.63
unidentified	1510.38±212.00	1285.05±214.39	676.39±420.67	1327.85±324.27	1406.30±71.31

Values are means of 3 triplicates per dietary treatment.

FM, fish meal diet (control diet); SBM60, high soybean meal diet with 600 g/kg fish meal protein replacement and without LF supplementation; LF2, LF6, and LF10 were added 2, 6, and 10 g/kg LF in SBM60 diets, respectively.
